# Biosensor with Microchannel for Broadband Dielectric Characterization of Nanoliter Cell Suspensions up to 110 GHz

**DOI:** 10.3390/bios14070327

**Published:** 2024-06-30

**Authors:** Wen Sun, Jianhua Wang, Jin Chen, Xiwei Huang, Xin Rao, Jiangtao Su, Yuqiao Huang, Boyu Zhang, Lingling Sun

**Affiliations:** 1 Key Laboratory of RF Circuits & System of Ministry of Education, School of Electronics and Information, Hangzhou Dianzi University, Hangzhou 310018, China; sunwen@hdu.edu.cn (W.S.); wangjianhua@hdu.edu.cn (J.W.); chenjin272@hdu.edu.cn (J.C.); huangxiwei@hdu.edu.cn (X.H.); raoxin1992@hdu.edu.cn (X.R.); jtsu@hdu.edu.cn (J.S.); 2 Zhejiang Provincial Key Lab of Large-Scale Integrated Circuits Design, School of Electronics and Information, Hangzhou Dianzi University, Hangzhou 310018, China; 3 Cancer Institute, The Second Affiliated Hospital, Zhejiang University School of Medicine, Hangzhou 310009, China; 22018271@zju.edu.cn; 4 Institute of Translational Medicine, Zhejiang University, Hangzhou 310029, China; 5 School of Biomedical Engineering, Shanghai Jiao Tong University, Shanghai 200240, China; boyuzhang@sjtu.edu.cn

**Keywords:** biosensor, dielectric constant, label-free, microchannel, cell suspension, cell capture structure

## Abstract

Cell dielectric property measurement holds significant potential for application in cell detection and diagnosis due to its label-free and noninvasive nature. In this study, we developed a biosensor designed to measure the permittivity of liquid samples, particularly cell suspensions at the nanoliter scale, utilizing microwave and millimeter wave coplanar waveguides in conjunction with a microchannel. This biosensor facilitates the measurement of scattering parameters within a frequency domain ranging from 1 GHz to 110 GHz. The obtained scattering parameters are then converted into dielectric constants using specific algorithms. A cell capture structure within the microchannel ensures that cell suspensions remain stable within the measurement zone. The feasibility of this biosensor was confirmed by comparison with a commercial Keysight probe. We measured the dielectric constants of three different cell suspensions (HepG2, A549, MCF-7) using our biosensor. We also counted the number of cells captured in multiple measurements for each cell type and compared the corresponding changes in permittivity. The results indicated that the real part of the permittivity of HepG2 cells is 0.2–0.8 lower than that of the other two cell types. The difference between A549 and MCF-7 was relatively minor, only 0.2–0.4. The fluctuations in the dielectric spectrum caused by changes in cell numbers during measurements were smaller than the differences observed between different cell types. Thus, the sensor is suitable for measuring cell suspensions and can be utilized for label-free, noninvasive studies in identifying biological cell suspensions.

## 1. Introduction

Microwave dielectric property is an inherent characteristic of substances, enabling the identification of samples based on their dielectric properties [1]. This method offers noninvasive, label-free, and real-time monitoring capabilities [2], finding applications across diverse fields such as food quality assessment [3] and monitoring the dielectric properties of biological tissues [4,5,6], including the discrimination between malignant and healthy tissues [7]. Extensive research has focused on measuring the macroscopic dielectric constant, often utilizing large waveguide structures [8,9], resonators [10,11,12,13,14], and coaxial probes [15], among others. At the micro level, microwave dielectric properties are employed to investigate intermolecular interactions, cell activity [16], cell state monitoring [17], and single cell detection [18,19,20,21,22] Traditionally, cell classification and analysis rely on optical detection systems, employing fluorescence staining techniques combined with microscopy and flow cytometry [23] which, though effective, are invasive, labeled, and costly for sample testing [24].

Microwave technology, with its label-free and noninvasive detection attributes, holds significant promise in cell recognition and monitoring [25]. Presently, most studies on cell impedance spectra focus on the KHz–GHz frequency range [26], with dielectric characteristics predominantly in the radio frequency and microwave frequency bands [12,27]. For instance, the species and activity of yeast particles were measured within the 0.1 GHz–8 GHz band range using microstrip sensors [16], revealing distinctions in membrane and activity states. Furthermore, a combination of microwave and optical sensing in the 2 GHz–3 GHz range successfully differentiated between cells and polystyrene particles [28], enabling the monitoring of cell capacitance changes post-chemical treatment. These sensors, equipped with narrow-gap sensing electrodes and integrated microchannel, have demonstrated the ability to detect individual cells within nanoliter volumes [29] measuring dielectric constants across a frequency range of 0.2~25 GHz. Impedance spectra analysis of cells within the 9 KHz~9 GHz range has been conducted using the double-shell model, proposing a comprehensive cell model including membrane, cytoplasm, and nucleus equivalent circuit models [17]. Coplanar waveguide (CPW) transmission lines are increasingly favored as sensing electrodes for small-volume liquid permittivity due to their open electric field, compact structure, simple manufacture, and ease of microchannel integration [30,31,32,33]. Nonplanar microwave sensors, characterized by their closed structure and low loss, exhibit high-quality factors and measurement sensitivity, but their large size and limited compatibility with microfluidic systems pose challenges in accurate liquid sample measurement.

Research in the millimeter wave band remains limited, yet holds promise due to the compatibility of sensor sizes with cell dimensions and the ability of millimeter waves to penetrate cell membranes. Variations in water and ion content across intracellular and extracellular fluids, cell membranes, and nuclei determine differences in millimeter wave dielectric properties, reflecting internal cellular information. Hence, the dielectric properties of cells in the millimeter wave band merit exploration. Advancements in micro–nano and microfluidic technologies facilitate accurate measurement of cell dielectric properties. To address challenges in detecting cell suspensions at the nanoscale, a sensor with a microchannel for measuring frequencies up to 110 GHz is proposed. This sensor integrates coplanar waveguide transmission structures with microchannel featuring cell capture structures, ensuring controlled cell numbers during each measurement. Characterizing the dielectric properties of cell samples at nanoliter volumes holds potential for cell-specific research, precision therapy, and the study of bioelectromagnetic effects and health in complex electromagnetic environments.

## 2. Materials and Methods

### 2.1. Sensor Design and Manufacture

**Figure 1:**
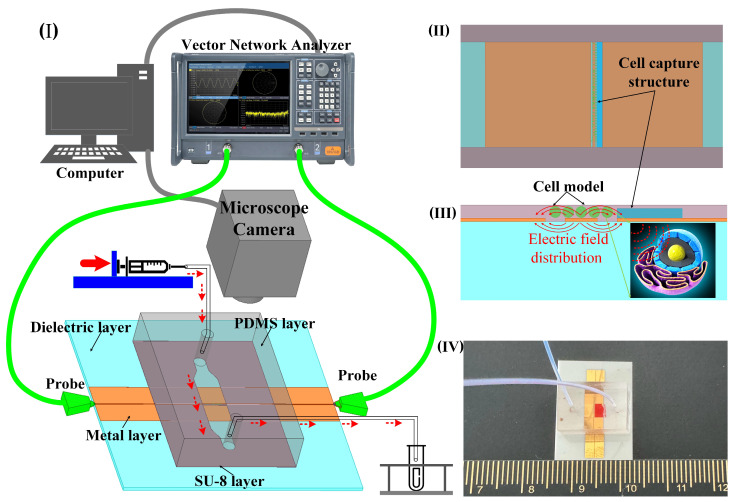
The sensor measurement architecture and function diagram. (**I**) 3D layered sensor with microchannel structure and test architecture schematic. (**II**) Schematic simulation of cell capture structure and cell model; the width of the microchannel is 2 mm, the height of the capture structure is 15 µm, and the width is 0.1 mm. (**III**) The diagram of electric field distribution of sensor and cell model. (**IV**) Photo of the structure size of the sensor made by micro–nano process; the overall size of the sensor is 20 mm × 20 mm, the width of the CPW core wire is 50 µm, the gap width is 30 µm, and the width of the single-side ground is 2 mm.

To achieve wide-band, noninvasive measurement considering the fluid nature of the liquid sample, a coplanar waveguide (CPW) serves as a sensor to determine the dielectric constant. This is based on alterations in the transmission characteristics of the electromagnetic wave as it traverses the sample. The measurement setup comprises a vector network analyzer (VNA), biosensor with microchannel, microinjection pump, microscope, and computer (Figure 1I). The sensor consists of a dielectric substrate, CPW metal sensor, and a microchannel. It is employed for assessing the scattering parameters of liquid samples in microchannels across coplanar waveguides. This sensor is suitable for noninvasively measuring the dielectric properties of biological liquid samples, such as cell suspensions and highly active protein solutions. The device operates within the wide band range of 1 GHz–110 GHz, with the dielectric constant derived through an appropriate algorithm.

In the analysis of liquid samples, the region between the source line of the CPW and the ground where the electric field is strongest is the region of primary concern. For cell suspensions, it is necessary for the cells to be precisely located within this sensitive area. Despite the random nature of cell positioning due to the fluid suspension, a cell capture structure is implemented to ensure cells remain within this critical area. This structure consists of a wall positioned at the edge of the CPW gap, measuring 15 μm in height and 5 μm below the microchannel surface (Figure 1II). This arrangement obstructs the cells while permitting the free flow of the liquid. In the simulation software Ansys19.0, the cells were replaced with medium particles of the same size. The electric field distribution around the CPW and the modeled cell indicates that sensor sensitivity is maximized when a cell occupies the CPW gap (Figure 1III). This configuration facilitates the penetration of electromagnetic waves through the cell membrane, enabling the detection of internal dielectric properties. To optimize broadband transmission capabilities, a ceramic, known for its low-loss properties, is chosen as the substrate for the CPW. The microchannel is fabricated from SU-8 photoresist, chosen for its thickness of 20 μm (Figure 1IV).

**Figure 2:**
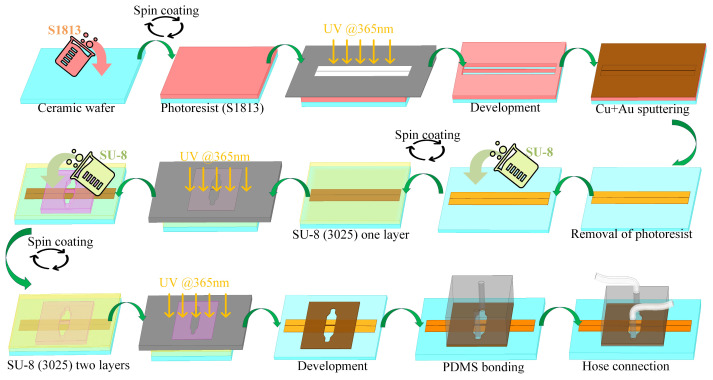
Sensor fabrication process flow chart.

The manufacture and assembly of the CPW sensor with an integrated microchannel involves three key components (Figure 2). The initial stage entails the preparation of the CPW metal layer. The second stage involves fabricating the microchannel using SU-8, and the third stage focuses on bonding this SU-8 layer to polydimethylsiloxane (PDMS). The metal layers are created through a series of steps that include coating with photoresist, photoetching, image development, depositing metals such as copper (Cu) and gold (Au), stripping the photoresist, and, finally, establishing a CPW metal pattern on the ceramic substrate. Different CPW lengths are utilized to examine transmission characteristics using a de-embedding technique. SU-8 photoresist, known for its robust adhesion and stability relative to both media and metal surfaces, is used to construct the microchannel. Following the initial SU-8 layer exposure, a second layer of SU-8 is spin-coated and subsequently developed together with the first after completing the photoetching process. Post-fabrication, the substrate is segmented into individual sensors by laser cutting. Given the nonenclosed nature of the microchannel, composed of SU-8, the channel’s top is sealed with PDMS. This elastomer is bonded to SU-8 following plasma treatment and amination with aminopropyl triethoxysilane. The direct application of PDMS in the microfluidic channels can lead to bonding challenges with the metal surface, which may cause leakage. To address this, silicone hoses are inserted at the inlet and outlet ports of the PDMS, facilitating controlled liquid sample delivery into the sensor’s microflow system.

The cured flat PDMS is precisely cut to the desired dimensions of 15 mm × 10 mm. Next, the PDMS substrate is accurately perforated at predetermined locations. Both the SU-8 microchannel and PDMS are thoroughly cleaned with deionized water and dried using either clean air or nitrogen. Subsequently, the bonding surface of the PDMS is treated with oxygen plasma to transition its surface from hydrophobic to hydrophilic properties. Additionally, the bonding surfaces of the SU-8 microchannel and PDMS are treated with an aminopropyl triethoxysilane (APTES) aqueous solution at a concentration of 3–5%. The treatment involves immersing the components in the APTES solution for 15–20 min, then pressing them together before heating on a hot plate at 90 °C for 10–20 min to complete the bonding process. This method, which does not necessitate the use of heavy weights on the chip, ensures high controllability, stability, and repeatability, resulting in a leak-resistant microchannel.

### 2.2. Preparation of Cell Samples

**Figure 3:**
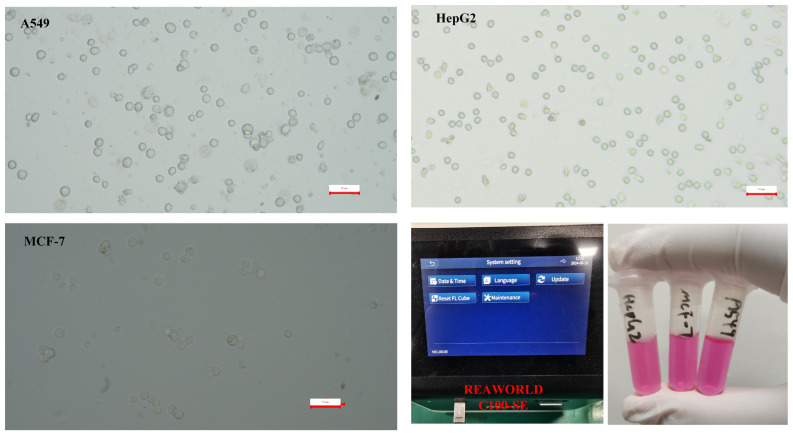
Micrograph of prepared tumor cell suspension sample and photos of prepared cells and cell counter operating interface.

First, lab-cultured tumor cells (A549, MCF-7, HepG2) undergo enzymatic digestion. Enzymatic digestion is a crucial step in cell culture and handling, particularly for cell revival and subculturing. Enzymatic digestion effectively separates cells, making them into a single-cell suspension, which is crucial for subculturing or cell revival. Separated cells are easier to count and assess for viability, ensuring consistent cell numbers and condition for experiments. The process may alter intracellular ion concentrations, influencing dielectric properties. Briefly, the culture medium is removed via pipette. The cells are subsequently washed with PBS solution. Digestive enzymes are then added to the cells, which are agitated and allowed to stand for 2 min before being washed with culture medium and centrifuged at 500× *g* for 10 min. The concentration of cells is determined and further analyzed using a cell counter. The resuspended tumor cell sample is diluted to an appropriate concentration as the test sample. Microscopic images of the three cell types are presented (Figure 3). For the prepared tumor cell suspension samples, the cell concentration, viability, and average size parameters were measured using a cell counter. The model of the cell counter used is RWD C100-SE with the following specifications: Counting area: 2.15 mm × 1.62 mm; cell size range: 4–60 μm (optimal: 7–60 μm); cell concentration range: 10^4^–10^7^ cells/mL; cell counting time: within 9 s, as shown Figure 3. The concentration of the cell sample for analysis is quantified using a cell counter, as indicated in Table 1.

### 2.3. Dielectric Constant Inversion Algorithm

**Figure 4:**
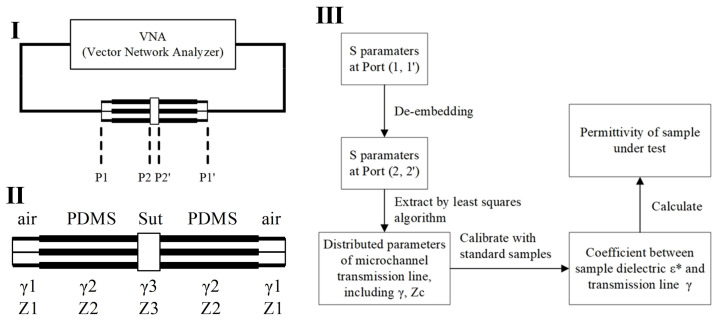
An algorithm for calculating the dielectric constant of a liquid sample from the scattering parameters measured by the sensor. (**I**) Block diagram of sensor and connection to vector network analyzer; (**II**) the transmission line distribution parameter model of the sensor; (**III**) dielectric spectrum extraction algorithm flow. The asterisk in ε* indicates that it is a complex physical quantity with both real and imaginary parts.

This sensor is based on a coplanar waveguide (CPW) transmission line, utilizing the transmission line method (TLM) to convert scattering parameters (S-parameters) into dielectric constants [30,34,35]. By measuring the S-parameters of the transmission line (CPW sensor) and employing the transmission matrix model of the transmission line, the dielectric constant can be calculated. The specific steps typically include the following: (1) Use a network analyzer to measure the S-parameters of the sensor containing the material under test over a specified frequency range. (2) Apply de-embedding algorithms to obtain the S-parameters of the sample under test. (3) Establish a mathematical relationship between the S-parameters and the dielectric constant based on the transmission matrix model of the transmission line. By employing optimization algorithms or analytical methods, the dielectric constant is extracted from the measured S-parameters. The transmission line method directly measures the S-parameters as a function of frequency, thus accurately reflecting the dielectric constant changes of the material at different frequencies. The scattering parameters are initially measured for the entire setup, which includes the sample-capturing transmission line segment, the capturing structure, and the transmission lines connecting to the probe (Figure 4I). To accurately determine the distributed parameters at the sample location, the sensor system is modeled as shown in Figure 4II. Using de-embedding techniques, the original scattering parameters obtained at the interface (P1, P1’) are recalculated to derive the scattering parameters at the sample interface (P2, P2’). Following this, a fitting method is employed to extract the distributed transmission line parameters specific to the sample segment. Since the scattering parameters, distributed transmission line parameters, and the sample’s dielectric constant are all frequency-dependent, the comprehensive procedure illustrated in Figure 4III is applied at each frequency point to determine the sample’s dielectric constant accurately. The de-embedding process and the fitting algorithm used to obtain the distributed parameters of the transmission line through the least squares method in Figure 4III are added as an appendix to the Supplementary Material. 

For more efficient data handling, the scattering matrix, derived from the transmission line measurements, is transformed into a transmission matrix. The specific transformation relationship between the scattering matrix and the transmission matrix is outlined as follows:

(1) $$\left[\begin{array}{cc} S_{11} & S_{12}\\ S_{21} & S_{22} \end{array}\right]=\left[\begin{array}{cc} T_{12}/T_{22} & T_{11}-(T_{12}T_{21}/T_{22})\\ 1/T_{22} & -T_{21}/T_{22} \end{array}\right]$$

(2) $$\left[\begin{array}{cc} T_{11} & T_{12}\\ T_{21} & T_{22} \end{array}\right]=\left[\begin{array}{cc} (-S_{11}S_{22}+S_{12}S_{21})/S_{21} & S_{11}/S_{21}\\ -S_{22}/S_{21} & 1/S_{21} \end{array}\right]$$

For transmission lines with characteristic impedances, the matrix for cascading impedance transformation of the transmission matrix is defined as follows:

(3) $$Q_{Zm}^{Zn}=\frac{1}{2Z_{m}}\left|\frac{Z_{m}}{Z_{n}}\right|\sqrt{\frac{R\left(Z_{n}\right)}{R\left(Z_{m}\right)}}\left[\begin{array}{cc} Z_{m}+Z_{n} & Z_{m}-Z_{n}\\ Z_{m}-Z_{n} & Z_{m}+Z_{n} \end{array}\right]$$

In Equation (3), the operation R(•) denotes taking the real part of the impedance. If the device exhibits a symmetrical structure, the overall transmission matrix for the transmission line is obtained by cascading the transmission matrices of each segment:

(4) $$T_{\mathrm{meas}}=Q_{Z1}^{50}T_{1}Q_{Z2}^{Z1}T_{2}Q_{Z3}^{Z2}T_{3}Q_{Z2}^{Z3}T_{2}Q_{Z1}^{Z2}T_{1}Q_{50}^{Z1}$$

In the equation, $T_{1}$, $T_{2}$, $T_{3}$, and $T_{\mathrm{meas}}$ represent the transmission matrices of the air segment, PDMS segment, sample segment, and the complete transmission line, respectively. The relationship between the transmission matrix of a transmission line with a specific length and its distributed parameters is expressed as

(5) $$T^{L}=\left[\begin{array}{cc} e^{-\gamma L} & 0\\ 0 & e^{\gamma L} \end{array}\right]$$

Knowing the distributed parameters $\gamma_{1}$ and $\gamma_{2}$ of the air segment and PDMS segment transmission lines, the T-matrix of the sample segment can be calculated using Equations (3)–(5). These parameters are determined through calibration using multiple uniform transmission lines, enabling the derivation of the S-parameters at interface (P2, P2’) from the S-parameters at interface (P1, P1’). 

To ascertain the distributed parameters $\gamma_{\mathrm{sample}}$ and $Z_{\mathrm{sample}}$ of the sample segment transmission line, the S-parameters are described as functions of distributed parameters (Figure 4II). The method of least squares is then employed to fit $\gamma_{\mathrm{sample}}$ and $Z_{\mathrm{sample}}$, ensuring that the calculated S-parameters under these distributed parameters have the smallest deviation from the S-parameters at the (P2, P2’) interface. The equivalent distributed parameters of the transmission line at the sample location are obtained by this method. Next, the relationship between the transmission line distributed parameters $\gamma_{\mathrm{sample}}$ and the sample’s dielectric constant $\varepsilon_{\mathrm{sample}}$ is established. Since the effective dielectric constant $\varepsilon_{\mathrm{eff}}$ of the transmission line is linearly related to the sample’s dielectric constant $\varepsilon_{\mathrm{sample}}$,

(6) $$\varepsilon_{\mathrm{sample}}=k\varepsilon_{\mathrm{eff}}+b,$$

and $\varepsilon_{\mathrm{eff}}$ is related to the transmission line’s γ as follows:

(7) $$\varepsilon_{\mathrm{eff}}={\left(\frac{c_{0}\gamma }{\omega }\right)}^{2},$$

then the coefficients k and b in the linear equation determining $\varepsilon_{\mathrm{eff}}$ and $\varepsilon_{\mathrm{sample}}$ can be obtained from two equations, thus obtaining the relationship between γ and $\varepsilon_{\mathrm{sample}}$. Finally, based on the obtained $\gamma_{\mathrm{sut}}$ from testing the sample, the dielectric constant $\varepsilon_{\mathrm{sut}}$ of the sample under test can be calculated.

The Keysight N1501A Dielectric Probe Kit utilizes the Open-Ended Coaxial Probe Algorithm to extract dielectric constants. Specifically, this algorithm calculates the complex dielectric constant of materials by measuring the reflection coefficient (S11) at the probe port and combining it with the probe’s electromagnetic field modes and geometric parameters. By fitting the measured data to the Debye model, the dielectric constant of liquids is extracted. This equipment is primarily suitable for low-frequency ranges and performs poorly at high frequencies. It also requires numerous fitting parameters, making the calculations complex. In comparison, the sensor employed here utilizes a high-frequency stable Elision model, capable of measuring dielectric spectra up to 110 GHz.

### 2.4. Construction of Test System and Measurement of Tumor Cells

**Figure 5:**
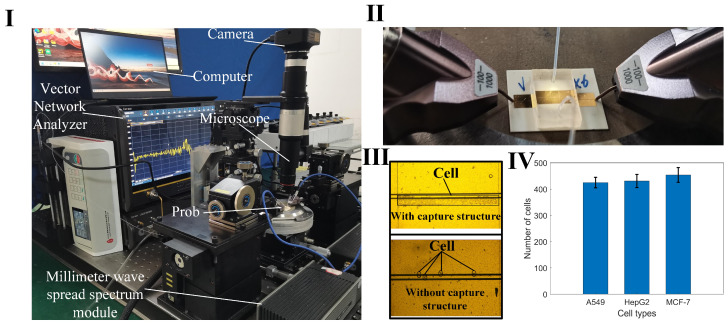
Measurement device and cell capture pictures. (**I**) A photograph of the setup featuring the sensor and associated measuring equipment; (**II**) Details of the connection between the probe (spaced at 100 µm between ground and signal) and the sensor during measurements; (**III**) A comparison of cell distributions in the sensing area, with and without the cell capture structure; (**IV**) Statistical data showing the variation in cell counts captured over ten measurement cycles.

The measurement apparatus includes a vector network analyzer (VNA), a spread spectrum module, a probe, a microinjection pump, a microscope, a camera, and a computer (Figure 5I). Positioned on a loading platform with negative pressure adsorption capabilities, the sensor is initially connected to the spread spectrum module via the probe (Figure 5II). One end of a hose is attached to the injector, while the other connects to a liquid waste recovery system. Upon completion of the setup, VNA parameters are configured, encompassing the measurement frequency range (1–110 GHz), port output power (−5 dBm), and scanning points (601). Calibration of the VNA is performed using a standard probe calibration technique. To increase the reliability of the measurements, we employed averaging techniques on the network analyzer during the measurement process, averaging multiple samples to reduce the impact of random noise. Setting a narrow intermediate frequency (IF) bandwidth can further reduce noise, with 100 Hz selected as the appropriate IF bandwidth as needed. Ensuring that the signal excitation level remains within the linear range of the network analyzer avoids nonlinear distortion caused by excessively high or low excitation levels. Subsequently, the test cell suspension sample is introduced into the sensor through the microflow injection pump. A syringe, containing 1 mL of the cell suspension and mounted on the pump, administers the sample at a flow rate of 1 µL/min. The control of cell numbers within the detection region is achieved through the combined regulation of the capture structure and the pressure from the injection pump. Firstly, the capture structure is designed with a height of 15 μm, which is close to the size of the cells, thus providing a blocking effect. Secondly, a 5 μm gap exists between the capture structure and the PDMS above it, allowing the liquid to pass through. By controlling the flow rate of the microfluidic injection pump, cells can be managed effectively: if the flow rate is too high, cells will flow past the capture structure; if the flow rate is too low, cells will accumulate excessively and not flow through. The optimal flow rate is set at 0.7 μL/min. Cells accumulate at the capture structure, and when the desired cell count is nearly reached, the injection pump is stopped. The residual pressure in the channel then causes any additional cells to push through the gap above the capture structure, ensuring cell count stability. This effect is demonstrated by comparing cell distributions in suspensions measured with sensors equipped with and without the capture structure (Figure 5III). After reaching a specified threshold, the amount of cell accumulation stabilizes. The microflow injection pump is carefully regulated to maintain a consistent cell count across each measurement, with data from 10 iterations showing stable cell accumulation (Figure 5IV). When the electromagnetic signal interacts with the interface of the cell sample, it modifies the transmission and reflection coefficients, which correlate to the sample’s dielectric constant. This constant is derived from the scattering parameters recorded by the VNA. The collected S-parameter data are then stored for further analysis.

## 3. Results and Analysis

### 3.1. Verification of Feasibility

**Figure 6:**
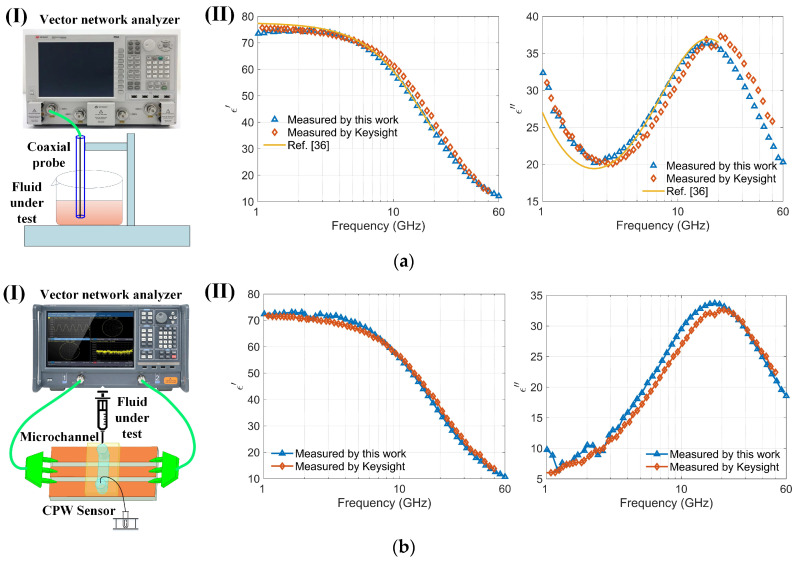
Comparison of the sensor with commercial instruments (Keysight N1501A): (**a**) (**I**) Schematic of the coaxial probe (Keysight N1501A) measuring a 150 mmol/L NaCl solution; (**II**) Comparison of the real and imaginary parts of the dielectric spectrum of a 150 mmol/L NaCl solution measured using both the Keysight N1501A and the sensor within the 1–60 GHz frequency range; (**b**) (**I**) Schematic of the sensor measuring a highly concentrated bovine serum albumin solution (BSA, 7.5%); (**II**) Comparison of the real and imaginary parts of the dielectric spectrum of a highly concentrated bovine serum albumin solution (BSA, 7.5%) measured using both the Keysight N1501A and the sensor within the 1–60 GHz frequency range.

The permittivity of the sample was determined by analyzing saved S-parameter data through a permittivity inversion algorithm. To validate the accuracy of both the sensor and the algorithm for characterizing dielectric constants, the sensor’s results were compared with those from a well-recognized commercial instrument, the Keysight N1501A, and with reported values in [36] (Figure 6a). Notably, the commercial Keysight equipment functions within a frequency range of 0.5 to 50 GHz, and, lacking higher frequency equipment, comparisons were made in the 1 to 60 GHz range to verify accuracy. The required volume of liquid for these measurements is substantial (several milliliters). The real and imaginary parts of the dielectric constant of the 150 mmol/L NaCl solution determined by the sensor were compared with the results measured using commercial Keysight software and those reported in the literature [36], as shown in Figure 6a. The maximum relative error observed between the sensor and the Keysight instrument measurements was less than 5%. Further, the dielectric properties of a highly concentrated bovine serum albumin (BSA) solution at 7.5% were examined using the sensor (Figure 6b). The results show that the real and imaginary components of the permittivity for the BSA solution are consistent with those measured using Keysight equipment. The sensor is capable of acquiring dielectric spectra of very small volumes (nanoliters) of cell suspensions. For the conductivity of a 7.5% BSA solution prepared in PBS, the PBS (phosphate-buffered saline) primarily determines the conductivity due to its ionic components, such as sodium chloride, potassium chloride, and phosphates [37]. The BSA itself does not significantly contribute to the conductivity [38]. Therefore, the imaginary part of BSA (7%) is smaller than that of NaCl solution as compared with Figure 6(aII,bII). Measuring the dielectric spectrum of high-purity and costly biological protein solutions with small sample volumes can substantially reduce costs and enable repeated experiments. Moreover, the sensor, operating up to 110 GHz and capable of measuring nanoliter-sized samples, offers significant advantages and potential for various applications. The measurement results confirm the sensor’s efficacy for determining the dielectric constants of biological liquids or cell suspensions.

**Figure 7:**
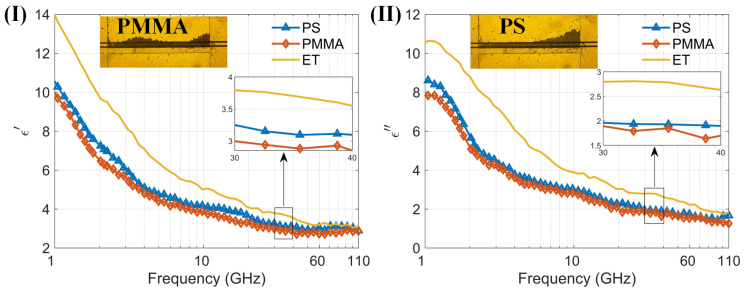
(**I**) The real part of permittivity of two kinds of particles PS and PMMA and background solution (anhydrous ethanol) measured by the proposed sensor; (**II**) The imaginary part of permittivity of two kinds of particles PS and PMMA and background solution (anhydrous ethanol) measured by the proposed sensor within the 1–110 GHz frequency range. (**I**) and (**II**) also show the microscopic images of PMMA and PS particle capture, respectively.

By measuring the dielectric spectra of particle suspensions, the sensor’s multifunctionality is further demonstrated. We measured small organic polymer particles made of two materials, mixed with anhydrous ethanol to form particle–ethanol suspensions, and tested their dielectric spectra using the sensor. The two types of organic particles are polystyrene and PMMA, both with diameters of 10 μm. Figure 7 shows the particles in the capture structure during testing and the dielectric spectra results, along with the microscopic images of particle capture. Since the particles do not deform, they do not easily pass through the capture structure, covering the entire detection area during measurement. It can be seen that the real part of the dielectric spectrum of ethanol and particle–ethanol suspensions decreases with increasing frequency in the 1–110 GHz range, while the imaginary part of the dielectric spectrum shows a decreasing trend with increasing frequency above 1.15 GHz, and an increasing trend in the 1–1.15 GHz range, with a maximum value around 1.15 GHz. The results of ethanol are consistent with the findings reported in reference [39]. Despite the particles filling the detection area, the liquid proportion remains high within this region. This is because the particle size of 10 µm is smaller than the total channel height of 20 µm, and there is still liquid present between the particles. Consequently, in the 1–110 GHz range, the overall trend of the dielectric constants of both particle–ethanol suspensions is similar to that of ethanol, dominated by the high dielectric constant of ethanol. The dielectric constants of the organic polymer particles are around 2.5–3. The measurement results indicate that the sensor can be used to measure the dielectric spectra of particle suspensions. Additionally, it has the capability to capture particles, providing a new method for stable measurement of the dielectric spectra of trace suspended particles.

### 3.2. Cell Suspension Measurement Results

**Figure 8:**
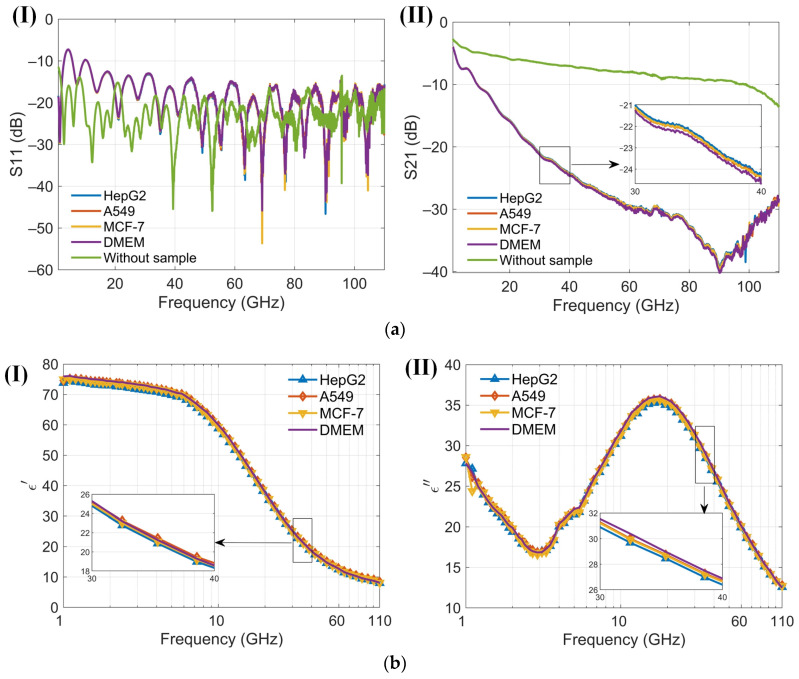
Results of four cell suspensions (1 GHz to 110 GHz). (**a**) (**I**) Reflection coefficients S11 of three kinds of cell suspensions and Dulbecco’s Modified Eagle Medium (DMEM) using the proposed sensor in the frequency range 1 to 110 GHz; (**II**) Transmission coefficients S21 of three kinds of cell suspensions and DMEM using the proposed sensor in the frequency range 1 to 110 GHz; (**b**) (**I**) The real part of the dielectric spectrum for three different cell suspensions and DMEM, as measured by the proposed senso; (**II**) The imaginary part of the dielectric spectrum for three different cell suspensions and DMEM, as measured by the proposed sensor.

**Figure 9:**
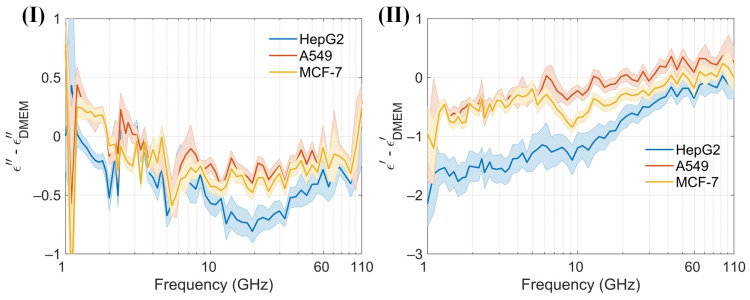
(**I**) and (**II**) Differences in the real and imaginary components of the dielectric spectrum between DMEM and the cell suspensions, quantified using the proposed sensor within the 1–110 GHz frequency range.

At low frequencies, the ions and electrolyzed amino acids in Dulbecco’s Modified Eagle Medium (DMEM), where amino groups are positively charged and carboxyl groups are negatively charged, significantly contribute to the dielectric constant because the ions can follow the alternating electric field, enhancing polarization [38]. As the frequency increases beyond the relaxation frequency, the electric field oscillates too rapidly for the ions to follow, resulting in a decrease in the dielectric constant. The dielectric constant of amino acids in the millimeter-wave range is typically low, around 2 to 4, because polar groups struggle to reorient quickly in the high-frequency electric field [40]. The cell membrane typically does not have a significant dipole moment. This is due to the symmetrical arrangement of lipid molecules in the bilayer, which cancels out the dipole moments of individual lipids. This symmetry results in a negligible net dipole moment for the entire membrane. An intact membrane exhibits dielectric dispersion associated with Maxwell–Wagner interfacial polarization. This phenomenon occurs at interfaces between materials with different dielectric properties and is characterized by a relaxation time [38]. The presence of water in DMEM significantly affects the dielectric properties of proteins. The dielectric constant of water molecules tightly bound to the protein surface is lower than that of free water. Overall, water contributes the most to the dielectric constant of DMEM [41]. Variations in the dielectric properties of tumor cells, influenced by differences in intracellular and extracellular fluids [42], cell membranes, nuclei, and the contents of water and ions, provide a foundation for noninvasive techniques in cell classification, identification, and detection [41,43]. The sensor is designed to measure the dielectric constant of a cell suspension within an effective range of about 13 nL, capable of detecting hundreds of cells. In the absence of a cell capture structure, the sensor typically detects between 1 and 50 cells, which may randomly distribute within the detection area. Furthermore, low cell counts can be challenging to detect without cell accumulation. Calibration of the sensor is conducted by measuring its no-load state and with deionized water before testing cells. Post-measurement, the DMEM is assessed, and the microchannel is cleansed and emptied using DMEM to maintain purity. Throughout the measurement process, the sensor configuration remains unchanged to eliminate potential errors from probe connections. The reflection coefficient (S11) and transmission coefficient (S21) from three different cell suspensions, as well as from the no-load sensor (air), are recorded. These data are used to calibrate the sensor and to compute the permittivity (Figure 8a). Permittivity values are subsequently calculated using a characterization algorithm. The observed trends in the dielectric constants of the cell suspensions and DMEM are consistent, albeit with distinct variations among different cells (Figure 8b). Both the real and imaginary parts of the dielectric constants for the three cell suspensions are generally lower than those of DMEM. To enhance the clarity of these differences, permittivity comparisons between the cell suspensions and DMEM are performed (Figure 9). A solid line represents the average of ten measurements, aiding in clear comparisons and minimizing drift errors in the measuring instruments. Notably, the real part of the permittivity of HepG2 cells is 0.2–0.8 lower than that of the other two cell types. This difference diminishes as frequency increases. Although the differences between the real and imaginary parts of the A549 and MCF-7 cells are modest, only 0.2–0.4, they are sufficient to enable effective differentiation. These findings support the application of this method in cell recognition and differentiation.

## 4. Conclusions

We propose a novel wideband sensor, which combines a microchannel featuring an integrated capture structure and a coplanar waveguide. This sensor operates within a frequency range of 1 GHz to 110 GHz and specializes in measuring the S-parameters of biological liquid samples. The dielectric constant is derived from these measurements using an advanced algorithm tailored for scattering parameter analysis. We validated the accuracy of our sensor by comparing its performance with that of established commercial Keysight instruments. Our study included measurements of BSA (7.5%) and tumor cell suspensions (A549, HepG2, MCF-7; 10^6^ cells/mL). This approach not only enables a precise comparison but also facilitates the effective removal of drift errors inherent in measuring instruments. The variance observed among the dielectric constants of the three tumor cell suspensions suggests a promising avenue for label-free and noninvasive cell identification. Additionally, the sensor is capable of detecting the dielectric constant of trace liquids and particle suspensions, presenting numerous potential applications. For example, it can be used to analyze the progress of chemical reactions, changes in solution composition, and molecular interactions. Specifically, in biochemistry, it can detect the behavior and interactions of biological macromolecules such as proteins and DNA. In the medical field, it can be used to analyze the components of biological fluids, such as blood and urine, aiding in disease diagnosis. Additionally, it can monitor the concentration and types of pollutants in water bodies, such as detecting harmful substances in industrial wastewater or analyzing organic content in natural water bodies, among other applications.

## Figures and Tables

**Table 1:** The results of cell suspension concentration using a cell counter.

Cell Type	Concentration (10^6^)	Average Diameter	Proportion of Living Cells
A549	1.38	18 µm	95%
HepG2	1.92	17.49 µm	91%
MCF-7	1.09	18.85 µm	86%

## Data Availability

Original data from this study are held by the author Wen Sun and can be obtained by request at sunwen@hdu.edu.cn.

## Supplementary Materials

The following supporting information can be downloaded at: https://www.mdpi.com/article/10.3390/bios14070327/s1, Figure S1: The de-embedding process and the fitting algorithm.
