# Supplementary figures and images for: Biosensor with Microchannel for Broadband Dielectric Characterization of Nanoliter Cell Suspensions up to 110 GHz
Source: Biosensors (Basel). 2024 Jun 30;14(7):327. doi: 10.3390/bios14070327 (PMC11274594; doi:10.3390/bios14070327)

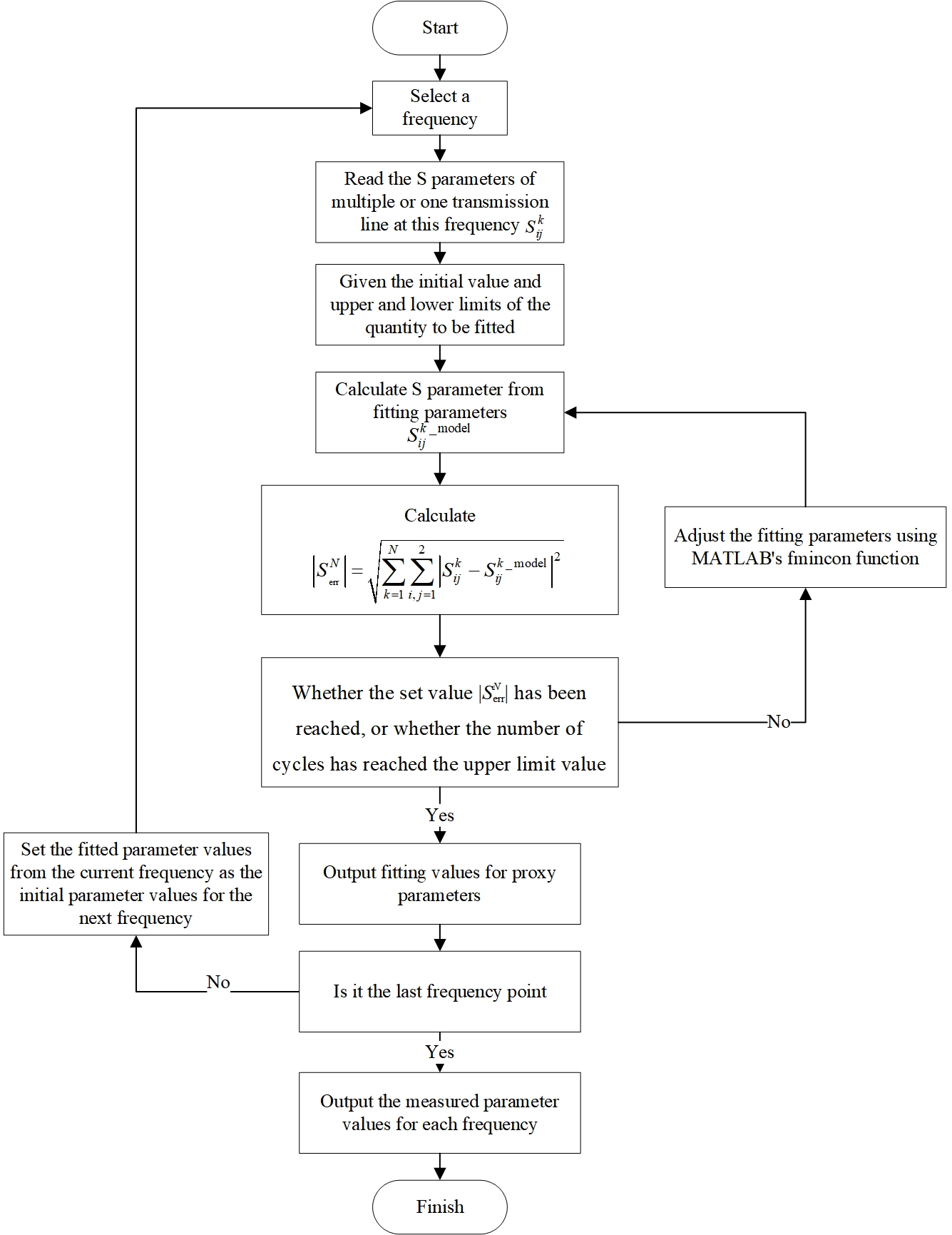

Supplement: Supplementary file 1 [file biosensors-14-00327-s001.zip › Figure S1 The de-embedding process and the fitting algorithm.tiff]
